# Simple and aneurysmal bone cyst: Aspects of jaw pseudocysts based on an experience of Brazilian pathology service during 53 years

**DOI:** 10.4317/medoral.21551

**Published:** 2016-12-06

**Authors:** Isadora-Luana Flores, Maria-Eduarda Hamilton, Elaine-de-Fátima Zanchin-Baldissera, Ana-Carolina Uchoa-Vasconcelos, Sandra-Beatriz Chaves-Tarquinio, Ana-Paula Neutzling-Gomes

**Affiliations:** 1 DDS, MSc, PhD. Department of Dentistry, Semiology and Maxillofacial Pathology area, Federal University of Juiz de Fora, Campus Governador Valadares, Governador Valadares, MG, Brazil; 2 Undergraduate Student. Pelotas Dental School, Semiology and Clinic Department, Federal University of Pelotas, Pelotas, RS, Brazil; 3 DDS, MSc, PhD. Pelotas Dental School, Semiology and Clinic Department, Federal University of Pelotas, Pelotas, RS, Brazil

## Abstract

**Background:**

Jaw pseudocysts are benign osseous lesions of unclear etiology. Among these, the simple bone cyst (SBC) and aneurysmal bone cyst (ABC) are intriguing bone pathologies still rarely studied together. This retrospective study aimed to present the long-term case series of patients with jaw pseudocysts focusing on the clinical, radiographic, and transoperative aspects.

**Material and Methods:**

A retrospective case series of patients with SBC and ABC was performed. Clinical, radiographic, and transoperative aspects of both pseudocysts were reviewed from the histopathological archives of 20,469 cases between 1959-2012. All descriptive data were summarized.

**Results:**

Of 354 (15.25%) bone pathologies, 54 cases of jaw pseudocysts were found, with 42 (11.86%) SBC and 12 (3.39%) ABC cases. For both lesions, most of the sample were young Caucasian women with an asymptomatic posterior mandible lesion with undetermined time of evolution and none trauma history. A unique radiolucent scalloped lesion presenting an empty cavity were also observed for both conditions. However, some atypical findings were found for SBC including: the expansion of bone cortical, tooth resorption, displacement of the mandibular canal, and recurrence. The absence of painful symptoms and the lack of classical blood-filled cavity were observed in some cases of ABC.

**Conclusions:**

The SBC and ABC are bone pathologies with few retrospective studies, no previous studies on the two conditions, varied nomenclature, and atypical aspects in some cases. Therefore, the knowledge of clinical, imaging, and transoperative features of such pseudocysts are clinically valuable as diagnosis hypothesis of radiolucent lesions of the jaws.

**Key words:**Simple bone cyst, aneurysmal bone cyst, pseudocysts, jaws.

## Introduction

By definition, a pseudocyst is a pathological cavity without lining epithelium and with clinical and radiographic similarities to true cysts, except for histopathological findings (1,2). Among the pseudocysts of maxillary bones, the Simple Bone Cyst (SBC), also known as Traumatic Bone Cyst, Hemorrhagic Bone Cyst, Solitary Bone Cyst, and Idiopathic Bone Cavity appears as a rare pathology (1,2). This entity accounts for only 1-2% of all pseudocysts/cysts in the maxillofacial region, and it is commonly found in the long bones (90%), humerus (65%), and femur (25%) (1,2).

In turn, the Aneurysmal Bone Cyst (ABC) is a pseudocyst similar to SBC in various aspects: most frequently found in the long bones (50%) and spine (20%), but rarely manifests in the jaw bones (2%) (3,4). However, this entity tends to have more aggressive clinical behavior than SBC (1-4). The etiology and pathogenesis of SBC are still uncertain and the literature suggests the presence of intraosseous hematoma caused by trauma, venous obstruction, disturbance of the local bone growth or changes in bone metabolism (2). These events may result in blood clot liquefaction and bone lysis (2). The most accepted theory for ABC etiology is a probable previous trauma resulting in blood accumulation inside the bone tissue (3,4).

Although the clinical, imaging, and histopathological aspects are well described, retrospective studies focused on the epidemiology and unusual clinical aspects of these jaw pseudocysts are still scarce. Therefore, based on the hypothesis of a greater incidence of these lesions in the jaw, this study aimed to conduct an epidemiological study of bone pathologies with emphasis on SBC and ABC from the files of an oral pathology service. A review of the most relevant classical aspects was also performed aiming to describe variations between these two entities in the English Literature.

## Material and Methods

The project was approved by the Ethics Committee in Research of the School of Dentistry, Federal University of Pelotas under protocol number 55/2013. A retrospective epidemiological study was conducted on the histopathological diagnosis report files of the Center of Diagnosis of Mouth Diseases (DCMD) of the Dentistry School, Federal University of Pelotas, Pelotas, Rio Grande do Sul, Brazil, between 1959-2012.

All histopathological diagnosis files were reviewed to identify SBC and ABC cases. The patients’ clinical charts were evaluated to obtain data about gender, age, race, anatomical location, clinical and radiographic findings, trauma history, type of treatment, recurrence, and follow-up. All data were tabulated and the results analyzed using descriptive statistics.

## Results

- Epidemiology

Of 20,469 records, this retrospective survey revealed 54 cases of pseudocysts of the jaws, less than 0.3% of the analyzed biopsy specimens. Of these, 42 (0.2%) were SBC and 12 (0.07%) were ABC. Considering only the bone pathologies (354 cases; 1.73%), the jaw pseudocysts accounted for 15.25% of the cases, with 11.86% and 3.39% of SBC and ABC lesions, respectively ( Table 1).

**Table 1:**
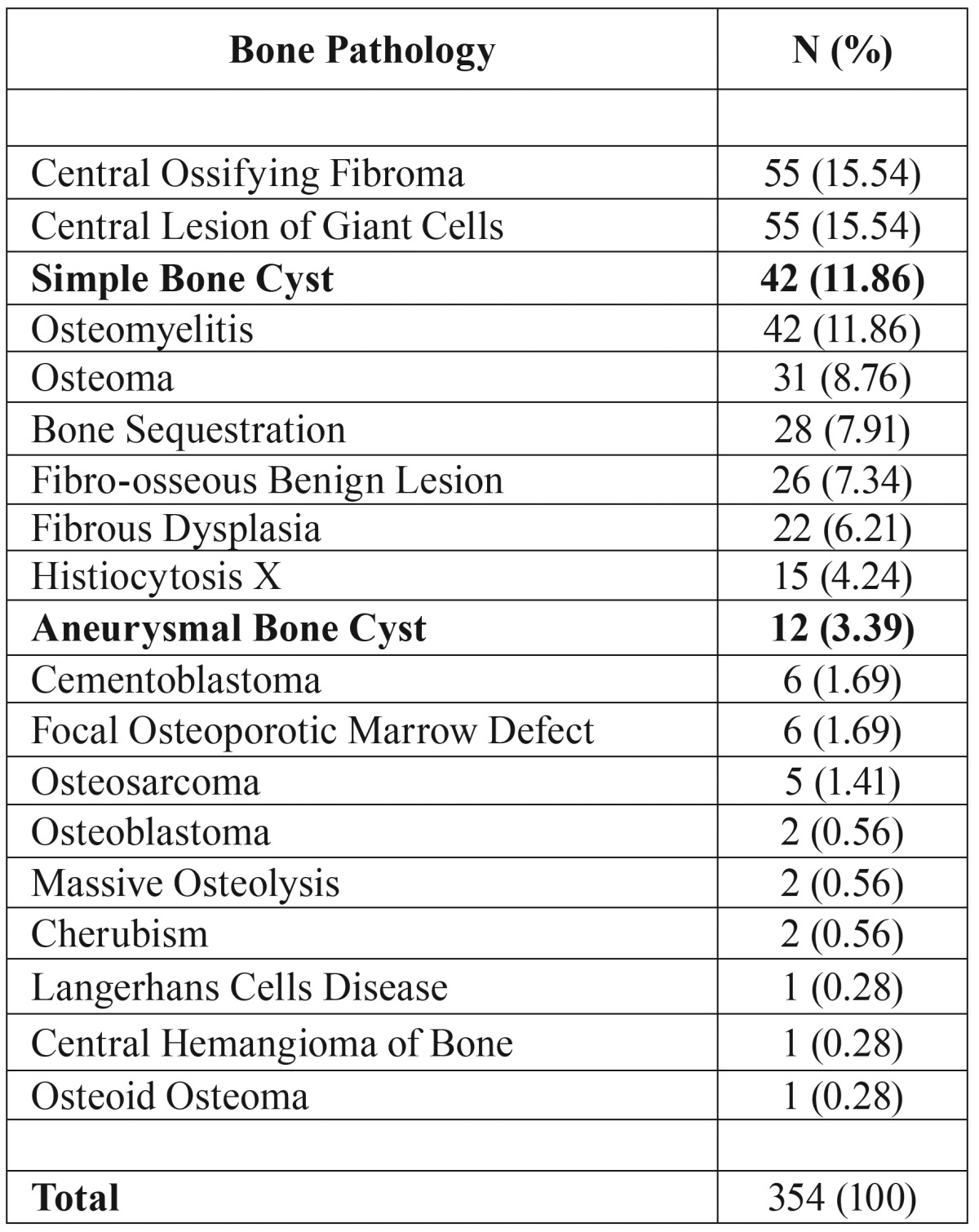
Epidemiological findings for SBC and ABC cases in relation to bone pathology diagnosed in the Center of Diagnosis of Mouth Diseases FO/UFPel between 1959 -2012.

*Main Clinical and radiographic features

- Simple Bone Cyst (SBC)

Of the 42 cases of SBC, 22 patients were female and 20 were male, with 37 cases diagnosed in Caucasian patients. The age varied between 7-66 years with mean age of 19.6 years and approximately 34 cases in the 2nd and 3rd decades of life. The history of previous trauma on the same area of the injury was reported only in 12 cases. All cases analyzed were located in the mandible, 29 cases in the posterior region, 10 cases in the anterior region, and about 3 cases in other locations. Facial asymmetry was identified only in 4 cases. In 6 cases, the time of evolution was <2 months and ≥1 year, but there were cases with only two weeks and cases with more than 4 years. However, the unknown evolution time and absence of symptoms were reported in most cases. In relation to clinical diagnosis, 27 cases had SBC as the first and only diagnosis hypothesis.

Additionally, of 42 cases, 39 had a radiographic description with 32 described as radiolucent lesion and only 7 cases as unilocular lesion. In 12 cases, dome-shaped and scalloped edges between the roots of the involved teeth were described. In 24 cases, the lesions were described as well-defined and in 2 cases as poorly-defined lesions. About the size of lesions on the radiographic images, there were variations of less than 1.5 cm to 8 cm. Five cases described expansion of cortical bone and 2 cases described resorption of the roots of teeth adjacent to the lesion. Noteworthy was the fact that in two cases displacement of the mandibular canal was reported.

- Aneurysmal Bone Cyst (ABC)

Of the 12 ABC cases analyzed, 10 patients were female and 2 males. All affected patients were Caucasian. The age variation was 10-36 years with mean age of 18.6 years; 9 cases occurred in the 2nd decade of life and 3 cases occurred in the 1st, 3rd, and 4th decade, respectively. A history of previous trauma in the region was reported in 5 cases. As for the location, 10 cases were in the mandible with 8 in the posterior region and 2 in the anterior. The maxilla was affected in 2 cases. The evolution time ranged from 1 month to 20 years (1 month in 2 cases): 1 year in 4 cases and more than 1 year of evolution in 6 cases. The facial asymmetry and no pain symptoms were reported in 9 cases with 2 patients presented mild discomfort and 1 patient reported persistent pain. In relation to clinical diagnosis, only in 1 case, ABC was considered as the unique clinical hypothesis. Additionally, 2 cases were firstly diagnosed as a central giant cell lesion (LCCG) and SBC, respectively.

**Figure 1:**
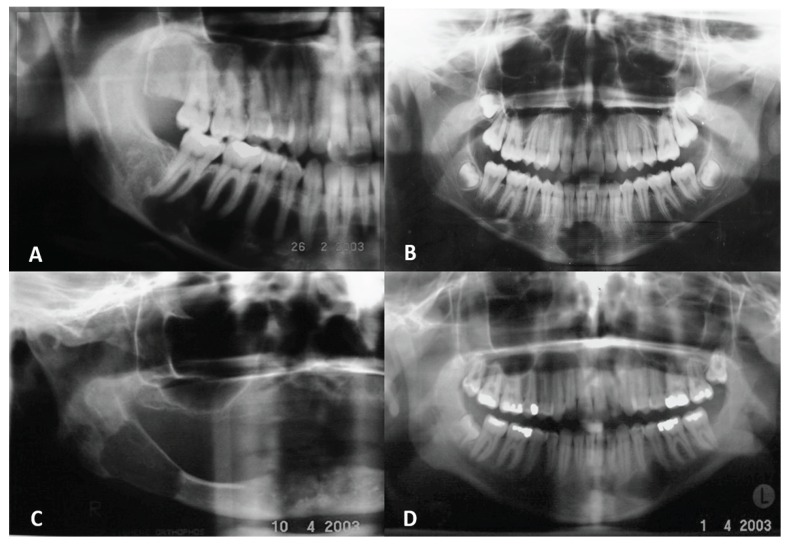
Panoramic radiographies of SBC and ABC. A. A classical aspect of SBC as a radiolucent dome-shaped lesion with scalloped edges between the tooth roots in the right posterior mandible. B. A well-defined radiolucent area in the anterior mandible is also the SBC presentation. C. A well-defined unilocular radiolucency in the posterior body and ramus of right mandible representing the ABC. Note the involvement of mandibular canal. D. A radiolucent unilocular lesion with sclerotic borders in the left mandible region are also the aspects found in the ABC lesions.

Radiographic findings of the ABC were obtained in 12 cases. Most lesions were described as radiolucent lesion with only 1 case reported with a radiopaque appearance of ground glass. Furthermore, only 3 cases were described as a multiloculated defect and in the other 9 cases, this information was not recorded, similarly to SBC. The contour of the lesions was recorded in only 4 cases, of which 1 case was described as a well-defined lesion and in 3 cases the limits were poorly-defined. In relation to the size, the lesions ranged from less than 1 cm to greater than 8 cm, this variable was described only in 5 cases. The expansion of cortical bone was observed in 5 cases, and the disruption of cortical bone observed in 4 of these cases. The main radiographic aspects of SBC and ABC are showed in the figure 1.

*Transoperative and histopathologic Features

- Simple Bone Cyst

Of the 42 cases of SBC, the aspiration biopsy revealed the presence of empty cavity in 25 cases, 11 cases with the presence of blood fluid, and 1 case with the presence of clear liquid. In the surgical approach, fragments described as “soft tissue” were found only in 5 cases.

- Aneurysmal Bone Cyst

**Figure 2:**
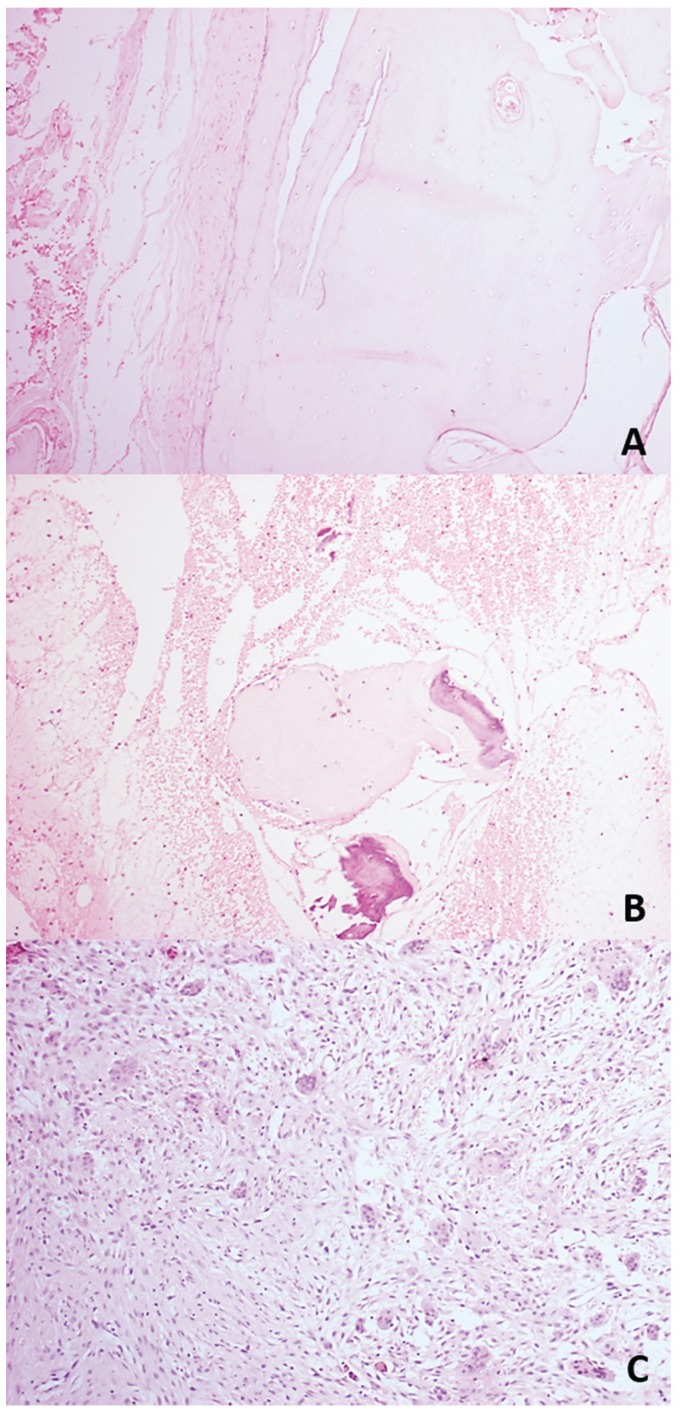
Histopathologic findings in the jaw pseudocysts. A. Scarce dense fibrous connective tissue neighboring the vital bone fragment can be observed in the SBC lesions (H&E, 40x). B. Fragments of vital bone surrounded by blood fluid with a loose fibrous connective tissue in the ABC. C. Dense fibrous connective tissue exhibiting a fibroblastic proliferation and multinucleated giant cells are also microscopical findings in the ABC lesions (H&E, 40x).

Of 12 ABC cases, 6 were diagnosed through excisional biopsies and 6 through incisional biopsies. The aspiration revealed 3 empty cavities, 2 cases with blood discharge, and in 1 case the presence of blood-yellowish liquid. The presence of thick capsule was observed in 1 case, and the description of “soft tissue” was also observed in 1 case. There was no transoperative description for the other cases. Some histopathologic aspects observed in SBC and ABC are showed in the figure 2. The most common clinical, radiographic and transoperative features of SBC and ABC are summarized in  Table 2.

**Table 2:**
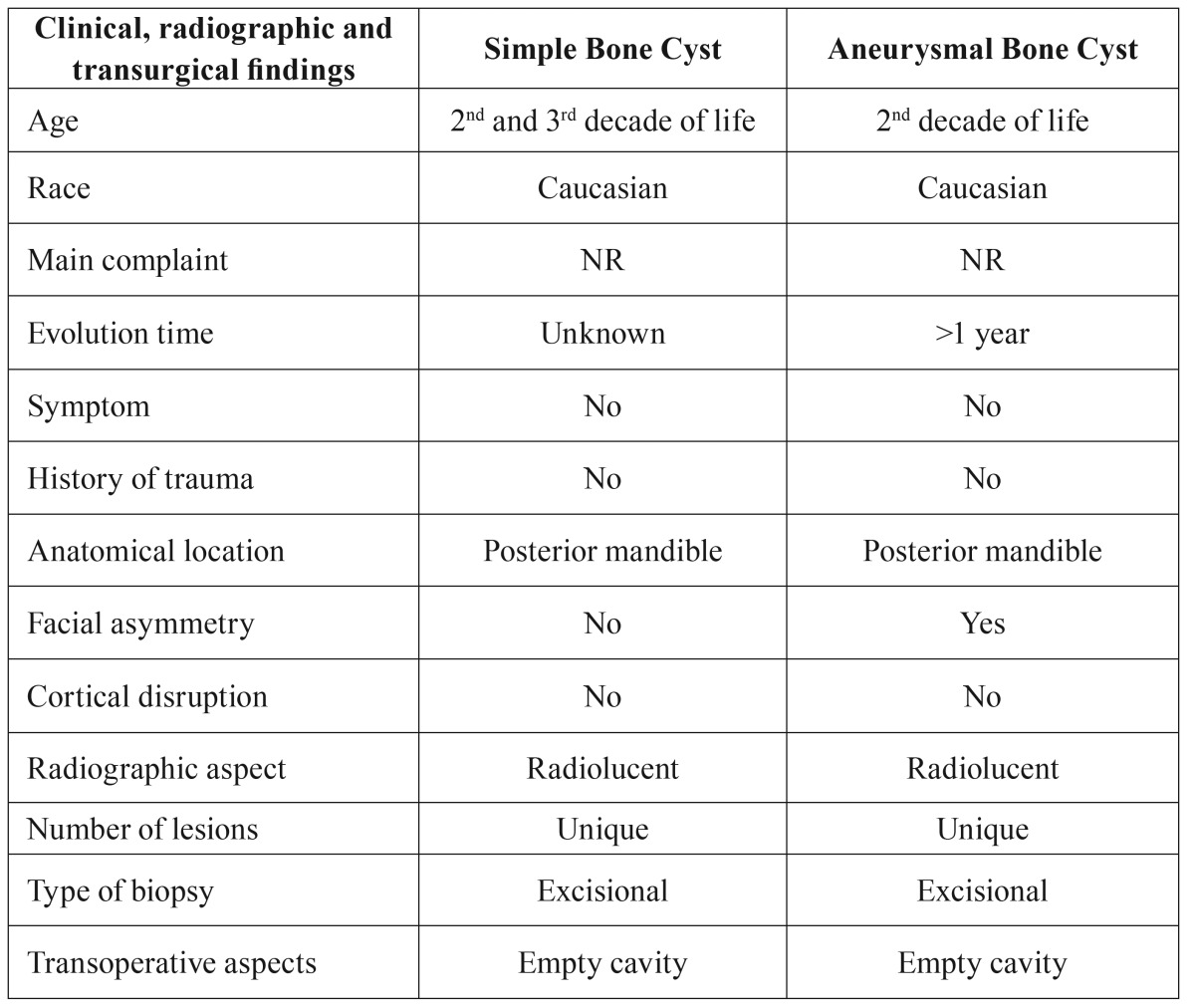
Main clinical, radiographic, and transoperative aspects of SBC and ABC according to the epidemiological survey of Diagnostic Center of Mouth Diseases, FO-UFPel the period 1959-2012.

## Discussion

The SBC and ABC are considered as rare entities in the jaw and the misuse of pseudocysts nomenclature made difficult to research the topic in PubMed/Medline database. Interestingly, no article was found on the search with the terms “pseudocysts, jaws, and Brazilian”. In addition, some authors erroneously defined such lesions as true non-odontogenic cysts or as other bone pathologies (5,6). Thus, a dynamic criteria established by this study was to evaluate the articles thoroughly, resulting from combination and individual search of the terms “SBC and ABC” associated with “jaws”. It should be noted that the variety of nomenclature, especially for SBC, with the inclusion of the names “traumatic bone cyst and idiopathic bone cavity” extended the results reached. However, manuscripts addressing both pseudocysts in the same study are still scarce in English Literature, mainly involving large series of cases (7).

In this context, the authors reported male as the most affected by SBC and no gender predilection for ABC (8-11). However, this study showed a higher prevalence of female for both bone entities. These findings may be associated with epidemiological variation in different parts of the world, given the retrospective studies of patients with significant sample published in English literature involving patients in different geographic areas (11-18). Furthermore, Brazilian studies with larger number of SBC and ABC cases are still scarce (15). However, multicenter studies are crucial to prove the epidemiological aspects found for these pseudocysts in the Brazilian population, especially because the present study involved an exclusive sample of an oral pathology service.

Interestingly, among the 354 cases of bone pathology diagnosed, SBC is the third most frequently entity (11.86%), followed by ABC (3.39%) ( Table 1). These results are contradictory with the literature findings in which the prevalence is around 1-2% for these lesions among cysts and pseudocysts of the jaws. However, this percentage can be explained by the exclusion of odontogenic lesions, especially cystic ones, which consequently would maintain the epidemiology of pseudocysts. Moreover, among the unusual clinical features observed in some SBC lesions, noteworthy is the expansion of cortical bone with resorption, the mandibular canal displacement, and recurrence. These findings are atypical and although the SBC is an innocuous lesion with no significance, some cases may have an unusual biological behavior with more aggressive aspects in its clinical course (19,20).

Concerning to ABC, the difficulty of including this pseudocyst as the initial clinical hypothesis involves not only the rarity of the injury, but also the presence of unusual clinical findings as the absence of pain symptoms in some cases and the presence of classical blood cavity in just 2 cases (10,11,14,15). Moreover, the association with other bone diseases and some microscopic features similar to LCCG, benign fibro-osseous lesions, and SBC may contribute to the difficulty of completing the histopathological diagnosis (21-24). Interestingly, the radiographic appearance of “ground glass” classic for fibrous dysplasia and the presence of empty bone cavity reinforce ABC misdiagnosis by other bone pathologies (24). Therefore, the correlation between clinical and imaging aspects and representative biopsy specimens are crucial to reach the final diagnosis of this entity.

Conclusions

SBC and ABC are bone pathologies with few retrospective studies, no previous studies on the two conditions, varied nomenclature, and atypical aspects in some cases. Therefore, knowledge of clinical, imaging, and transoperative features show applicable clinical value for the dentist due to the necessity of including such pseudocysts as diagnosis hypothesis facing of radiolucent lesions of the jaws.
